# Detection of Oxidative Stress Induced by Nanomaterials in Cells—The Roles of Reactive Oxygen Species and Glutathione

**DOI:** 10.3390/molecules26164710

**Published:** 2021-08-04

**Authors:** Jan Čapek, Tomáš Roušar

**Affiliations:** Department of Biological and Biochemical Sciences, Faculty of Chemical Technology, University of Pardubice, Studentska 573, 532 10 Pardubice, Czech Republic; Tomas.Rousar@upce.cz

**Keywords:** reactive oxygen species, oxidative stress, glutathione, nanotoxicity, cell injury, fluorescence probes

## Abstract

The potential of nanomaterials use is huge, especially in fields such as medicine or industry. Due to widespread use of nanomaterials, their cytotoxicity and involvement in cellular pathways ought to be evaluated in detail. Nanomaterials can induce the production of a number of substances in cells, including reactive oxygen species (ROS), participating in physiological and pathological cellular processes. These highly reactive substances include: superoxide, singlet oxygen, hydroxyl radical, and hydrogen peroxide. For overall assessment, there are a number of fluorescent probes in particular that are very specific and selective for given ROS. In addition, due to the involvement of ROS in a number of cellular signaling pathways, understanding the principle of ROS production induced by nanomaterials is very important. For defense, the cells have a number of reparative and especially antioxidant mechanisms. One of the most potent antioxidants is a tripeptide glutathione. Thus, the glutathione depletion can be a characteristic manifestation of harmful effects caused by the prooxidative-acting of nanomaterials in cells. For these reasons, here we would like to provide a review on the current knowledge of ROS-mediated cellular nanotoxicity manifesting as glutathione depletion, including an overview of approaches for the detection of ROS levels in cells.

## 1. Introduction

Molecular oxygen (O_2_) has a significant effect on numerous chemical reactions and biological processes. O_2_ reductions are one of the most critical electrocatalytic reactions that function in electrochemical energy conversion [1]. Free radicals contain an unpaired electron mostly bound to oxygen atoms. Conversely, the group of compounds named reactive oxygen species (ROS) also contains molecules without an unpaired electron, e.g., hydrogen peroxide [2,3]. Thus, the group of ROS also contains oxygen free radicals such as superoxide or hydroxyl, alkoxyl, peroxyl, and nitroxyl radicals [4,5]. The production of ROS is commonly linked with mitochondria, where the electrons are transferred through the respiratory chain to O_2_ forming water [6,7]. Mitochondrial ROS production depends on many factors such as the membrane potential of mitochondria [8], concentration of mitochondrial respiratory substrates, or a type of cells [9]. Mitochondria are the most important sources of superoxide and hydrogen peroxide in mammalian cells. The production of these ROS occurs mainly on the mitochondrial respiratory complex I and III [7,10]. In addition to mitochondrial complexes, ROS is also produced in mammalian cells by the participation of other enzymes such as flavoproteins [11] and other enzymes involved in nutrient metabolism [12]. As ROS plays important roles in the regulation of cell death processes, i.e., apoptosis [13] or necrosis [14,15,16], their pathological roles have been identified in a number of diseases including cancer and other age-related degenerative processes [17,18]. Given their deleterious effects, ROS production is usually finely tuned by ROS-scavenging systems [9].

Nanomaterials (NMs) exhibit great potential for use in the biomedical, optical, and electronic fields [19,20,21,22,23]. However, nanomaterials have been considered as potentially toxic due to their unique properties. They have extremely high surface-to-volume ratios, making them very reactive and catalytically active [24]. Their toxic potential in cells is also supported by their small size, enabling them to easily penetrate cell membranes [25]. TiO_2_ is one of the most commonly used nanomaterials in the chemical industry (e.g., cosmetics and pigments) [26]. In addition to white lead properties, TiO_2_ can be very active in photocatalytic reactions with organic compounds, providing the formation of ROS including ^●^OH, O_2_^●^^−^, H_2_O_2_ [27]. In addition to TiO_2_, other nanomaterials of different chemical compositions can produce ROS. The overview of NMs capable of ROS production is summarized in Table 1 including the lifetime.

Nanomaterials or nanoparticles (NPs) can expose transition metals on their surface, which can generate ROS through Fenton or Haber-Weiss reactions [44]. During these reactions, hydrogen peroxide is reduced in the presence of transition metals (Fe^2+^, Cu^+^) to form a highly active and toxic hydroxyl radical. Thus, the role of nanomaterials in ROS-mediated cell damage is significant and ROS production induced by NMs can lead to the modulation of various intracellular pathways, e.g., NF-κB, caspases, MAPK, etc., involving the activation of cell death processes [45,46].

In this study, we aimed to provide a recent and detailed view on ROS production induced by nanomaterials. The importance of our review can be also supported by the role of increased ROS levels that can lead to glutathione depletion and to the activation of cellular signaling pathways, resulting in changes in cellular metabolism, cell damage, or even in cell death.

## 2. Reactive Oxygen Species

### 2.1. Superoxide

**Figure 1:**
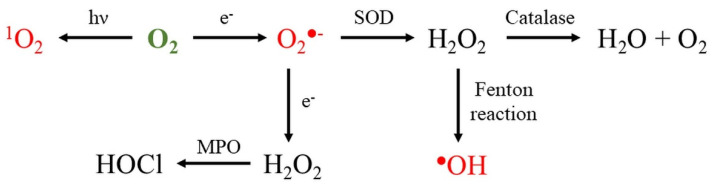
Formation of reactive oxygen species. Abbreviations: SOD = superoxide dismutase; MPO = myeloperoxidase; O_2_ = oxygen; ^1^O_2_ = singlet oxygen; O_2_^●^^−^ = superoxide; H_2_O_2_ = hydrogen peroxide; ^●^OH = hydroxyl radical; HOCl = hypochlorous acid; and *hν* = radiation. ROS colored in red are free oxygen radicals.

Superoxide radical is formed during enzymatic and non-enzymatic reactions in biological systems [1,47]. In atoms and molecules, paired electrons occur usually as antiparallel, which strongly limits the oxidation properties of O_2_. After one-electron reduction of molecular oxygen, the superoxide radical (O_2_^●^^−^) forms. This reaction is thermodynamically very unfavorable and the interaction of O_2_ with another paramagnetic center is important for overcoming spin restriction [48]. Although the reactivity of O_2_^●^^−^ is mild, the crucial role of superoxide is that it enables the formation of other ROS (Figure 1), playing important roles in the pathology of various diseases.

Superoxide radical (O_2_^●^^−^) is formed mainly in mitochondria and its reactivity with biomolecules is relatively low. Superoxide can be produced after the reaction of molecular oxygen with divalent metals catalyzing a single-electron reduction under their simultaneous oxidation (equation 1).

(1) $$O_{2}+{\;\mathrm{Fe}}^{2+}\;\to {\;O}_{2}^{.-}+{\;\mathrm{Fe}}^{3+}$$

Another formation can be catalyzed by enzymes including xanthine oxidase, lipoxygenase, or cyclooxygenase [49]. The superoxide radical may exist in two possible forms: either in the form of O_2_^●^^−^ at physiological pH or as a hydroperoxyl radical (HO_2_^●^) at low pH levels [50]. Hydroperoxyl radical penetrates better through phospholipid bilayers compared to the charged form O_2_^●^^−^ [28,51]. The superoxide radical may react with another superoxide radical to form hydrogen peroxide and O_2_ (equation 2). The reaction is catalyzed by the enzyme superoxide dismutase (SOD) [52,53]. A product of the dismutation reaction is H_2_O_2_ which becomes an important factor in the formation of the most reactive ROS, i.e., hydroxyl radical (^●^OH) [54].

(2) $$O_{2}+{\;O}_{2}^{.-}+2H_{2}O\;\overset{\mathrm{Cu},\;\mathrm{Zn},\;\mathrm{Mn}-\mathrm{SOD}}{\to }{\;H}_{2}O_{2}+{\;O}_{2}$$

The mitochondrial electron transport chain (ETC) has been attributed to the role as the main ROS generator in cells. When transporting electrons, some of the electrons from the ETC can reduce molecular oxygen to O_2_^●^^−^ [55]. The resulting O_2_^●^^−^ is rapidly dismissed by mitochondrial superoxide dismutase (Mn-SOD) forming H_2_O_2_ [56]. Mitochondrial ETC consists of several electron transporters (flavoproteins, proteins containing iron and sulfur, ubiquinone, and cytochromes) with redox potentials ranging from −0.200 to +0.600 V [57,58]. According to the respective redox potentials, the individual electron carriers are arranged in individual complexes of the respiratory chain I–IV. Electrons that are transported into the respiratory chain as reducing equivalents of NADH or FADH_2_ enter the ETC through mitochondrial Complexes I and II. Then, the electrons are transferred through ETC to Complex IV which reduces O_2_ to H_2_O. From the thermodynamical perspective, all these electron transport systems could transfer the electrons directly to O_2_ to form O_2_^●^^−^. However, there are only two major sites of the respiratory chain where ROS can be generated, i.e., at Complexes I and III [59,60].

In Complex I, a reaction occurs between O_2_ and the reduced form of the flavinmononucleotide (FMN), leading to production of O_2_^●^^−^. The amount of reduced FMN depends on the NADH/NAD^+^ ratio [61]. In Complex III, two specific binding sites for coenzyme Q10 are known, i.e., Qi and Qo. Superoxide production is located in Qo. When antimycin A is added as an inhibitor of the Qi site, O_2_^●^^−^ production increases [62], while the addition of a myxothiazole inhibitor for the Qo site decreases ROS production [63]. Under physiological conditions, the production of ROS in Complex III depends on the ∆Ψ. The rate of O_2_^●^^−^ formation may increase exponentially with increasing ∆Ψ. This directly correlates with the fact that due to ∆Ψ fluctuations, the transport of electrons from heme bL to heme bH slows down, which then increases superoxide generation [64]. 

#### 2.1.1. Role of Superoxide in Nanomaterial Toxicity

Damage to mitochondria and subsequent ROS leakage is a commonly accepted mechanism of nanoparticles toxicity. Damaged mitochondria release O_2_^●^^−^ into the inter-membrane space which can ultimately damage the cell [65]. Across different types of nanomaterials, their involvement in the ROS generation can be found. Far more often than in size, their possible cytotoxic effects are chemically dependent. Despite the similar size and crystal shape of ZnO NPs and SiO_2_ NPs, higher toxicity of ZnO NPs is observed, where cell viability is reduced and O_2_^●^^−^ generation is reduced, due to which glutathione (GSH) depletion occurs [29]. TiO_2_ nanoparticles generate O_2_^●^^−^ [30] both in solution and in cells, and intracellular O_2_^●^^−^ reduces the expression of histone deacetylase 9 (HDAC9), an epigenetic modifier [66]. Cellular internalization of TiO_2_ NPs has been shown to activate macrophages and neutrophils contributing to the production of O_2_^●^^−^ by the NADPH oxidase [67]. Oxidative stress induced by excessive O_2_^●^^−^ production is an important mechanism of the CuO NPs toxicity [31]. CuO NPs can enter HepG2 cells, where they are capable of inducing cellular toxicity by generating O_2_^●^^−^ leading to GSH depletion [68]. Activation of mitogen-activated protein kinases (MAPKs) and redox-sensitive transcription factors was demonstrated, suggesting that MAPK pathways and redox-sensitive transcription factors could be major factors of CuO NPs toxicity [69].

Analysis of mouse fibroblasts and human hepatocytes revealed that an increase in ROS levels induced by Ag NPs is accompanied by a reduction of mitochondrial membrane potential, release of cytochrome c into the cytosol, JNK activation, and translocation of Bax to mitochondria [32]. After exposure to Ag nanoparticles, GSH depletion occurs in liver cells, which is directly related to ROS production [70]. Ag NPs appear to induce DNA damage through a mechanism involving ROS production.

#### 2.1.2. Methods for the Detection of Superoxide

##### MitoSox

**Figure 2:**
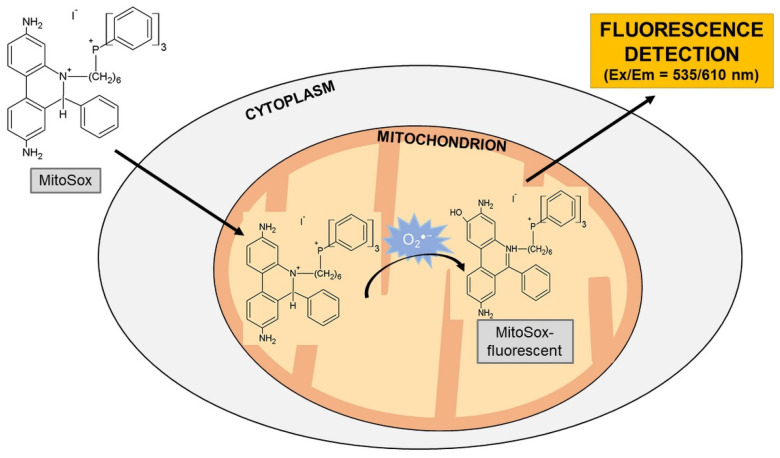
Detection of superoxide using MitoSox fluorescent probe. Abbreviation: O_2_^●^^−^ = superoxide.

Hydroethidium (HE) is a selective O_2_^●^^−^ detection probe (Figure 2) that reacts very rapidly to changes in O_2_^●^^−^ concentration, forming a red fluorescent product with 2-hydroxyethidium cation (2-OH-E^+^). Hydroethidine is a reduced form of ethidium that can be oxidized to ethidium in cells. The resulting ethidium intercalates nucleic acids and significantly increases its fluorescence, emitted at 610 nm (excitation = 535 nm) [23,71].

A new hydroethidine analog was synthesized for the purposes of O_2_^●^^−^ detection, which is produced in mitochondria. This analog carries a charged triphenylphosphonium residue (Mito-HE; Mito-Sox Red). As the phosphonium residue is positively charged and surrounded by three lipophilic phenyl groups, it penetrates very easily through cell membranes, mainly through the inner mitochondrial membrane [72]. After they cross the cell membranes, they accumulate in mitochondria depending on the negative ∆Ψ [73]. Importantly, redistribution of MitoSox from mitochondria is dependent on decreasing ∆Ψ based on various stimuli, which may not be ROS. For this reason, the use of MitoSox is a semi-quantitative test. Very important is the fact that MitoSox is transferred from mitochondria to the cytoplasm. Here, the supply of nucleic acids is higher and the increasing fluorescence is independent to mitochondrial ROS production, which may distort the results of individual measurements. The formation of MitoSox oxidation products in mitochondria may result in changes of values, which may reduce the passage of other MitoSox molecules into the mitochondria and generally affect measurements due to decreased MitoSox and ROS concentrations that are not produced by breathing chain breakage. The fluorescent product emits radiation at 580 nm with excitation at 540 nm [74,75,76].

##### 1,3–Diphenylisobenzofuran

The 1,3-diphenylisobenzofuran (DPBF) probe is a molecule that, when incorporated into liposome phospholipids, acquires fluorescent properties. It is used for the detection of O_2_^●^^−^ and ^1^O_2_. After reaction with oxygen radicals, it produces a decrease of fluorescence, thus the fluorescence rates correlate inversely with increasing concentrations of O_2_^●^^−^ and ^1^O_2_ [77,78]. The reaction of DPBF with ROS such as singlet oxygen, hydroxyl, alkoxy and alkyl peroxy radicals gives 1,2-dibenzoylbenzene. In contrast, only reaction with H_2_O_2_ produces 9-hydroxyanthracen-10-(9H)-one. This product can be detected using fluorescence spectroscopy, NMR spectroscopy, or HPLC [79]. 

### 2.2. Hydroxyl Radical

The hydroxyl radical is a neutral form of the hydroxide ion. It belongs among the most reactive ROS because it can react with a variety of organic and inorganic compounds including DNA, proteins, and lipids, resulting in serious cell damage. The hydroxyl radical may be formed as a product of the Fenton or Haber–Weiss reaction [80,81,82,83].

The Fenton reaction is based on the reaction between H_2_O_2_ and Fe^2+^. Iron is an essential component of many proteins involved in the transport or metabolism of oxygen due to its ability to undergo cyclic oxidation and reduction. Iron has to be present for the ongoing synthesis of iron-containing proteins. As such, it can directly lead to the formation of free radicals, which can cause cellular damage of large extent. The reaction of Fe^2+^ with H_2_O_2_ produces an oxidized form of iron (Fe^3+^), as well as ^●^OH and OH^−^ (Equation (3)).

(3) $${\mathrm{Fe}}^{2+}+{\;H}_{2}O_{2}\;\to {\;\mathrm{Fe}}^{3+}\;\left[H_{2}O_{2}^{-}\right]\;\to {\;\mathrm{OH}}^{-}+{\;}^{●}\mathrm{OH}$$

(4) $$O_{2}^{.-}+{\;H}_{2}O_{2}\to O_{2}+{\;\mathrm{OH}}^{-}+{\;}^{●}\mathrm{OH}$$

Another possible reaction to form ^●^OH is the Haber–Weiss reaction. In this reaction, less reactive O_2_^●^^−^ and H_2_O_2_ react with each other (Equation (4)). As in the case of the Fenton reaction, very toxic ^●^OH is formed. Very unfavorable thermodynamic conditions are applied to this reaction, in which the rate constant in the aqueous solution is close to zero. The presence of a transition metal catalyst is required to ensure the reaction. The iron atom serves as the catalyst. Both reactions produce highly reactive ^●^OH, which ultimately severely damages cells [84,85,86,87]. The Fenton reaction can be used to induce apoptosis in cancer cells, where ^●^OH is formed on a copper ion [88,89].

#### 2.2.1. Role of Hydroxyl Radical in Nanomaterial Toxicity

TiO_2_ and ZnO NPs are widely used in cosmetics and industry [22]. Under the influence of UV radiation, ZnO NPs generate reactive oxygen species such as ^●^OH or H_2_O_2_, causing GSH depletion [33,90]. The rate of ^●^OH generation and the total photocatalytic activity depends on the physical properties of the nanomaterial used, e.g., TiO_2_ NPs [34]. Cu NPs play an important role as a cofactor in a number of enzymes such as cytochrome c oxidase [91]. However, they exhibit significant toxicity and can induce ROS production, including largely reactive ^●^OH. Copper can catalyze electron transfer (Cu^2+^ and Cu^+^). This can give rise to O_2_^●^^−^ reduction to H_2_O_2_ in cells, leading to GSH depletion [35]. Other particles that induce ^●^OH production include Fe_3_O_4_ [92], silica nanoparticle [93], and silver nanoparticles [94].

#### 2.2.2. Methods for the Detection of Hydroxyl Radical

Terephthalic acid (TA) can be hydroxylated in presence of ^●^OH to give the highly fluorescent product 2-hydroxy-TA [95]. TA has a configuration of two carboxylate anion (COO^−^) side groups attached to a six-carbon ring at positions *1* and *4* to form a structurally symmetrical compound. Reaction of ^●^OH with any of the four unsubstituted carbons will form only one hydroxylated product, 2-hydroxy-TA (2-OH-TA). TA is non-fluorescent, whereas 2-OH-TA is highly fluorescent. Neither TA nor 2-OH-TA is present in tissues physiologically. In addition, none of them is known to be involved in cellular functions, thus they exhibit no cellular toxicity [96].

Fluorogenic spin probes can be used to detect ^●^OH. Their signal can be detected both fluorometrically and using EPR spectroscopy. The rhodamine nitroxide probe is a non-fluorescent substance reacting quantitatively with ^●^OH (Ex/Em = 560/588 nm) [97]. 

The HKOH-1 probe was designed for better uptake and longer retention in cells. The HKOH-1 probe has excellent sensitivity, selectivity, and extremely rapid turn-on response toward ^●^OH in live cells in both confocal imaging and flow cytometry experiments [98]. 

### 2.3. Singlet Oxygen

Singlet oxygen (^1^O_2_), the highest energy state of molecular oxygen, has been extensively studied to oxidize toxic persistent organic contaminants [99]. Singlet oxygen is a highly reactive form of oxygen. It is produced during photochemical reactions or even physiologically in the respiratory chain of mitochondria. In excitation, molecular oxygen is excited to the first state (1∆g) and then to the higher excited state (1∑g). In the first excited state, O_2_ has two counter-spin electrons in a π orbital, while in the second excited state, O_2_ has one counter-spin electron in two π orbitals [100,101]. The first excited state is highly reactive. 1∆g ^1^O_2_ is also produced physiologically, e.g., in the activation of neutrophils and macrophages [102,103]. It is a highly potent oxidizing agent that can cause fatal damage of DNA [104] or cell death [105,106].

Singlet oxygen reacts with several biological molecules including DNA, RNA, lipids, sterols, and especially proteins [107]. Amino acid residues of proteins can react with ^1^O_2_ by direct chemical reaction or physical quenching. Physical quenching causes de-excitation of the singlet state of oxygen proved in proteins through the interaction with tryptophan residues [108].

#### 2.3.1. Role of Singlet Oxygen in Nanomaterial Toxicity 

**Figure 3:**
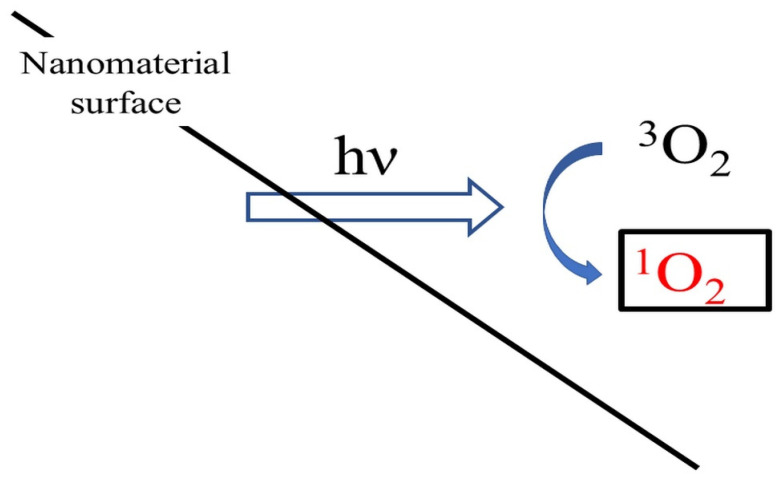
Generation of ^1^O_2_ in a photocatalytic reaction on the TiO_2_ surface. Abbreviations: ^3^O_2_ = molecular oxygen; ^1^O_2_ = singlet oxygen; and hν = radiation.

Reactive oxygen species are formed by the reaction of photoinduced binding electrons with oxygen molecules. After the release of photoinduced electrons, valence band holes are formed on the surface of TiO_2_ NPs that cannot oxidize water [109]. Another type of ROS that occurs during photocatalytic reactions on the surface of TiO_2_ NPs is ^1^O_2_ (Figure 3) [41]. Nanomaterials that can induce singlet oxygen production also include Ag NPs [42]. Nanomaterials-bound generation of ^1^O_2_ can be also used in the treatment of tumors [43]. An activatable system has been developed that enables tumor-specific ^1^O_2_ generation, based on a Fenton-like reaction between linoleic acid hydroperoxide (LAHP), tethered on FeO NPs and Fe^2+^ ions released from FeO NPs under acidic pH conditions [43]. After increased production of ^1^O_2_ in cells, the intracellular concentration of GSH decreases [110,111,112].

#### 2.3.2. Methods for the Detection of Singlet Oxygen

The DPAX-1 fluorescent probe (9-[2-(3-carboxy-9,10-diphenyl)-anthryl]-6-hydroxy-3H-xanthen-3-one) has been used to detect ^1^O_2_ forming endoperoxide as a reaction product. The probe is based on 9,10-diphenylanthracene (DPA), conjugated to fluorescein. The high quantum yield and wavelength of the excitation radiation are suitable for biological applications [113]. The DMAX 9-[2-(3-carboxy-9,10-dimethyl)anthryl]-6-hydroxy-3H-xanthen-3-one has been also used to detect ^1^O_2_. The DMAX probe reacts much more specifically and faster with ^1^O_2_ compared to the DPAX-1 probe [114].

Other approach for singlet oxygen detection are amino-functionalized nanoparticles covalently linked to Singlet Oxygen Sensor Green^®^ (SOSG) which is an anthracene-fluorescein dye. The fluorescence of the SOSG molecule is inhibited by photoinduced intramolecular electron transfer. When anthracene is endoperoxidized in the presence of ^1^O_2_, the electron transfer is blocked and fluorescein self-fluorescence is restored [115].

### 2.4. Hydrogen Peroxide

Hydrogen peroxide is formed directly through SOD-catalyzed dismutation from superoxide [116]. It belongs among ROS but it is not a free radical. The relatively long lifespan and size of H_2_O_2_ allows it to pass through cell membranes to different parts of the cell, which facilitates signaling reactions [117]. It causes cell damage at concentrations higher than 100 nM. Concentration of H_2_O_2_ in the range of 1–10 nM acts physiologically in the process of redox signaling [116]. It does not cause direct DNA damage but DNA damage is ensured due to ^●^OH presence, which arises from H_2_O_2_ in the presence of transition metal ions [118]. Enzymes eliminating H_2_O_2_ include catalase, glutathione peroxidase, and peroxiredoxins [119].

In peroxisomes, the main metabolic process producing H_2_O_2_ is the β-oxidation of fatty acids through acyl-CoA-oxidase. Other enzymes involved in the formation of ROS include urate oxidase [120], D-aspartate oxidase [121], or xanthine oxidase [28].

#### 2.4.1. Role of Hydrogen Peroxide in Nanomaterial Toxicity

Most nanomaterials that induce the production of O_2_^●^^−^ also induce the production of H_2_O_2_. In a study [36], colorectal cancer cells were exposed to polystyrene NPs (20 and 40 nm) with two surfactants (amino and carboxylic acid). After the exposure of cells to polystyrene NPs, a decrease in cell viability was observed and the induction of the apoptosis process was reduced by decreased H_2_O_2_ production by catalase. In another study [37], the authors observed a decrease in intracellular GSH concentration after the exposure of cells to 8 nm Au NPs. Subsequently, it was found that there was a decrease in mitochondrial membrane potential (∆Ψ) and cell apoptosis deepened after 48 h of incubation of cells with Au NPs. Then, a decreased mitochondrial GSH concentration and increased H_2_O_2_ production were observed. Other nanomaterials capable of induction of H_2_O_2_ formation are e.g., TiO_2_ NPs [38], ZnO NPs [39], and Ag NPs [40]. 

#### 2.4.2. Methods for the Detection of Hydrogen Peroxide

##### 2′,7′-Dichlorodihydrofluorescein

**Figure 4:**
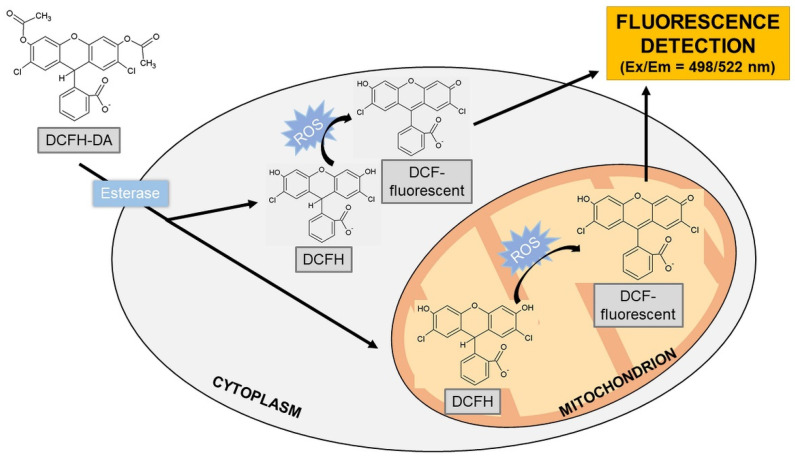
Detection of hydrogen peroxide using a probe DCFH-DA. Abbreviations: DCFH-DA = 2′,7′-dichlorodihydrofluorescein diacetate; DCFH = 2′,7′-dichlorodihydrofluorescein; DCF = 2′,7′-dichlorofluorescein; and ROS = reactive oxygen species.

The 2′,7′-dichlorodihydrofluorescein (DCFH) probe is a specific indicator of the presence of H_2_O_2_. The diacetate form of DCFH (DCFH-DA) has been used to detect ROS in cells due to its ability to penetrate cell membranes. Two acetate groups are hydrolyzed by intracellular esterases after DCFH-DA transfer into cells. Then, the presence of peroxidases is important for the oxidation of DCFH by H_2_O_2_. Other agents capable of oxidizing DCFH include hematin or cytochrome c [122,123] which may increase the fluorescence of the probe without any H_2_O_2_ production [124]. DCFH can be also oxidized with H_2_O_2_ in the presence of Fe^2+^ but this is most likely due to the formation of ^●^OH. In contrast, O_2_^●^^−^ is unable to oxidize the DCFH probe [125]. In the presence of visible light or ultraviolet radiation, a DCF photoreduction can occur (Figure 4). The fluorescent product exhibits fluorescence at 522 nm (excitation at 498 nm).

The oxidation of the probe produces a semichinone radical (DCF^●−^) that, when reacted with O_2_, gives rise to O_2_^●^^−^. Dismutation of O_2_^●^^−^ produces H_2_O_2_ that then artificially increases the oxidation of DCFH. The oxidation of DCFH results in the formation of a fluorescent product DCF exhibiting strong fluorescence. However, this reaction can increase the fluorescence intensity of the DCF product and give false-positive results [126,127,128]. In the case of the measurement of ROS production in tested nanomaterials, the form of DCFH-DA has been mostly used in ZnO_2_ NMs [33,129,130,131,132] and TiO_2_ NMs [133,134,135,136].

##### Amplex Red

**Figure 5:**
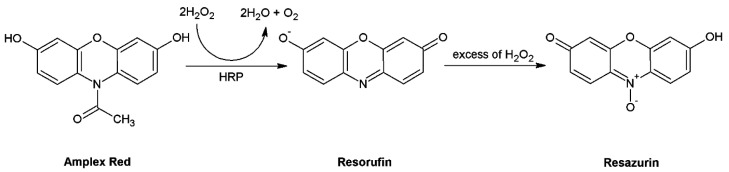
Oxidation of Amplex Red to a fluorescent (resorufin) and non-fluorescent (resazurin) product. Abbreviation: HRP = horseradish peroxidase.

Amplex Red (N-acetyl-3,7-dihydroxyphenoxazine) is a non-fluorescent molecule that can be specifically oxidized by H_2_O_2_ in the presence of horseradish peroxidase (HRP) to the highly fluorescent resorufin product (Figure 5), EX/EM 563/587 nm [137]. At excessive H_2_O_2_ concentrations, the fluorescent product resorufin can be further oxidized to non-fluorescent resazurin [138]. Amplex Red reacts with H_2_O_2_ stoichiometrically. It can also be used for the detection of O_2_^●^^−^ in a mixture with SOD converting O_2_^●^^−^ to H_2_O_2_. The background fluorescence during the measurement is very low and the fluorescent product is very stable. These features increase the sensitivity of the measurement. Significant loss of fluorescence may be due to the oxidation of resorufin to the non-fluorescent resazurin product that can be catalyzed by HRP [139,140].

##### HyPer Ratiometric Sensor

The H_2_O_2_ concentration can be measured using the expression of a HyPer genetically encoded ratio sensor. HyPer consists of the bacterial H_2_O_2_-sensitive transcription factor OxyR, fused to the circular fluorescent protein YFP. Cysteine oxidation of the OxyR moiety induces a conformational change that results in an increase in YFP fluorescence intensity excited at 500 nm and a decrease in YFP emission excited at 420 nm. This reversible change can monitor the intracellular concentration of H_2_O_2_ [141].

##### Pentafluorobenzenesulfonyl Fluoresceins

Perhydrolysis of acyl resorufins is a reaction that acts as a fluorescent indicator for the determination of H_2_O_2_. This method is based on deprotection rather than oxidation, which enables the fluorescence of resorufin and fluorescein. The selectivity of this method for H_2_O_2_ detection is higher compared to DCFH. For the above reasons, pentafluorobenzenesulfonyl fluoresceins have been proposed as selective fluorescent probes for H_2_O_2_ detection. Importantly, sulfonates are more stable to hydrolysis than esters. Fluoresceins have high fluorescence yields and the pentafluorobenzene ring increases the reactivity of sulfonates with H_2_O_2_ [142].

##### Europium Ion

The method is based on the binding of Eu^3+^-tetracycline [Eu (tc)] linked to propanesulfonic acid (MOPS) in an aqueous solution to H_2_O_2_. After binding, a strongly fluorescent complex ([Eu (hp) (tc)]) is formed (λ_EX/EM_ = 390-405 /616 nm). The increase in fluorescence is up to 15x after H_2_O_2_ binding and it is strongly dependent on the pH value. The increase in fluorescence is most pronounced at the physiological pH environment. The fluorescence of the probe [Eu (tc)] is not affected by ammonium, chloride, sulphate, or nitrate ions. However, citrate and phosphate can interfere with the assay [143].

##### Homovanilic Acid

Recently, homovanillic acid (3-methoxy-4-hydroxyphenylacetic acid) has been increasingly used instead of scopoletin for H_2_O_2_ detection in mitochondria. In contrast to the fluorescent scopoletin indicating the presence of H_2_O_2_ by a fluorescence decrease, homovanillic acid becomes a fluorescent through H_2_O_2_-induced oxidation in the presence of HRP [144]. The product of this reaction is a highly fluorescent dimer 2,2′-dihydroxy-3,3′-dimethoxydiphenyl-5,5′-diacetic acid [145]. In the following Table 2, an overview of all described fluorescent probes for ROS detection are summarized.

## 3. Role of Reactive Oxygen Species Induced by Nanoparticles in Cell Signaling

**Figure 6:**
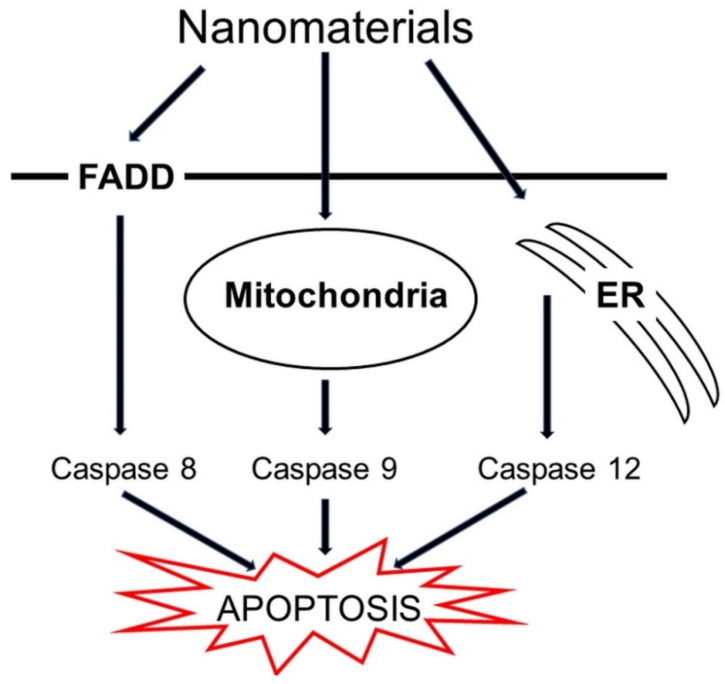
Possible pathways of induction of apoptosis by nanomaterials in cells. Abbreviations: ER = endoplasmatic reticulum and FADD = FAS-associated death domain protein.

Nanomaterials are capable of interfering with cell signaling pathways. Recently, three main pathways participating in the apoptosis process have been identified (Figure 6). The first pathway is the direct NMs occupation of the FADD receptor. The second pathway is the modulation of the function of mitochondria in the presence of NMs and the third is the localization of NMs pacting in the endoplasmic reticulum. All of these pathways converge upon caspase activation, thereby the mitochondria produce higher levels of ROS, increase production of Bid protein, and activate Bax or Bak1 proteins, which can ultimately lead to organelle damage, DNA cleavage, and cell death [146]. 

The dynamic and rapid nature of ROS signaling is the result of ROS production and removal. The balance between the production and removal of ROS is balanced due to their interaction. This causes rapid changes in ROS levels [147]. ROS play an important role in activating many cellular proteins and factors, e.g., NF-κB, MAPK, Keap1-Nrf2-ARE, or PI3K-Akt [148,149].

The NF-κB family is a family of transcriptional proteins consisting of five members, i.e., NF-κB1, NF-κB2, RelA, RelB, and c-Rel [150]. The activation of the transcription factor NF-κB involves signal-dependent degradation of phosphorylated inhibitors such as IκBα. The mechanism of NF-κB activation by H_2_O_2_ [151] or O_2_^●^^−^ [152] is different from the activation in the presence of cytokines or mitogens. Serines 32 and 36 play a key role in the activation of NF-κB by cytokines, while tyrosine residues 42 and serine/threonine in the PEST domain of the IκBα protein play a key role in the activation by H_2_O_2_ [153]. H_2_O_2_ activates IκBα kinase without subsequent serine phosphorylation of IκBα. In contrast, H_2_O_2_, similar to TNF, induces serine phosphorylation of the p65 subunit of NF-kB, leading to its nuclear translocation [154]. Nanoparticles participate directly in the activation of the factor NF-κB through increased ROS production which was confirmed by the translocation of the high-mobility group box 1 (HMGB1) protein from the nucleus to the cytoplasm observed in cells after exposure to silica nanoparticles [155]. Subsequently, HMGB1 binds to the TLR4 receptor; this complex regulates the expression of the myeloid differentiation factor and activates the NF-κB-signaling pathway.

In eukaryotic cells, signaling by MAPK kinases is very important. Various MAPK pathways can be activated by different stimuli. Ultimately, activated MAPK pathways coordinate gene transcription activation, acting in the regulation of protein synthesis, cell cycle, cell death, and cell differentiation [156]. The MAPK cascade is composed of three distinct signaling modules, i.e., the c-Jun N-terminal kinase cascade, the p38 MAPK cascade, and the extracellular signal-regulated kinase ERK [157]. Several cellular stimuli activating ROS production can also activate MAPK activation itself [158]. For instance, MAPK kinases can be activated by H_2_O_2_ [159]. MAPK activation occurs by activating growth factor receptors in several cell types [160]. Another mechanism of MAPK activation by ROS is the inactivation of the MKP protein by its oxidation [161]. The physiological FEM protein keeps the MAPK signaling pathway inactive. In addition to the activation of MAPK, the JNK pathway is also activated during the oxidation of the FEM protein [162]. A number of studies have demonstrated the activation of a variety of kinases by ROS, including ASK1 [163], MEKK1 [164], c-Src [165], and EGFR [166]. These activated kinases ultimately can activate the MAPK cascade [167]. Cerium oxide particles have been shown to activate ROS production and to reduce SOD and glutathione peroxidase activities. This results in increased phosphorylation levels of p38 MAPK as well as ERK1/2 and JNK [168]. The nanoparticles that can damage cells through p38 MAPK activation are silica NPs [169,170], polystyrene NPs [171], and TiO_2_ NPs [172]. Conversely, the exposure to Au [173] and iron oxide [174] NPs causes the osteogenetic differentiation through the activation of relevant genes by p38 MAPK.

The tumor suppressor protein p53 induces apoptotic cell death in response to oncogenic stress. Malignant progression is dependent on the loss of p53 function by mutations in the TP53 gene itself or defects in signaling pathways. Phosphorylation of p53 regulates the ability to activate the expression of apoptotic target genes [175]. Overexpression of p53 transactivates a number of p53 genes. Many of these genes encode redox active proteins including enzymes (quinone oxidoreductase and proline oxidase) generating ROS. Ultimately, this regulation of ROS production leads to oxidative stress that can induce apoptosis [176]. Increasing the intracellular concentration of ROS leads to the activation of the p38 protein, which increases the expression and transcriptional activity of p53 [177]. The p53 protein transcriptionally activates the PUMA gene encoding two proteins, PUMA-α and PUMA-β, of similar activity. These proteins bind to Bcl-2 and integrate into the mitochondria, where they induce the release of cytochrome c [178,179,180].

Last but not least, ROS activate the JNK kinase pathway, which plays an important role in the apoptosis process [4,181]. During intracellular ROS production, there is a permanent activation of JNK [182]. This is due to the inactivation of MAPK phosphatases (FEM) by oxidation of their catalytic cysteine in the presence of intracellularly accumulated H_2_O_2_. Expression of catalytically inactive FEMs prolongs JNK activation [183].

## 4. Current Trends in the Evaluation of Nanotoxicity In Vitro

The number of studies focusing on nanotoxicity testing has been growing very rapidly in the last two decades. The cause of that can be also found in the perpetual production of new nanomaterials for its following use in industry or medicine. Conversely, especially in medicine, nanomaterials raise some concerns regarding their cytotoxicity or biocompatibility. Thus, a number of scientific projects have been assessing the toxicity of the selected nanomaterials and creating the risk management framework for the use of nanomaterials in medical applications [184].

Recent studies on nanotoxicity have been using basic assays for the evaluation of cell function changes, e.g., cell viability, membrane integrity, and enzyme activities measurements. To estimate the oxidative status in cells, the levels of antioxidants can be measured using a number of methods. In addition to the most frequently used methods, other approaches have been used to characterize the cellular nanotoxicity recently. These methods include scanning electron microscopy [185], liquid cell transmission electron microscopy [186], atomic force microscopy [187], and hyperspectral and laser confocal microscopy applied to cell-nanoparticles interactions [185]. All these microscopic methods are very sensitive and specific, which allows for a very detailed description of the function state of the cells after nanomaterials treatment. To understand the toxicity of nanomaterials, we need to develop new and innovative methods that will provide us with information about the changes in the intracellular environment after exposure to nanomaterials. In addition, there is a need to develop methods that are fast, robust, and combine several biological tests. In contrast to conventional assays using lipophilic fluorescent probes detecting ROS levels, a nanoelectrode has been developed to study the toxicity of magnetic nanoparticles. The nanoelectrode is composed of individual platinum nanoelectrodes with a cavity at the tip. It is part of an upright microscope and is used to measure intracellular ROS [188].

A further topic of interest in nanotoxicity testing is the use of newly developed relevant biological models. In comparison to two-dimensional (2D) cultured cell lines, those new biogical models ought to provide accurate predictions of nanomaterials effects in vivo. Thus, some new scientific studies described the use of pulmonary fibrosis models [189], organ on-chip technology bridging the differences between 2D in vitro and three-dimensional (3D) in vivo models from skin, the lung, and the liver [190,191], or on-chip placenta models [192]. Despite advanced organ on-chip models, a number of concerns have to be solved to ensure the comparability to living systems in obtained outcomes [193].

## 5. Conclusions

Currently, nanotechnology is considered to be one of the most attractive research topics due to its huge application potential and commercial impact. Due to the large number of newly manufactured nanomaterials, it is necessary to evaluate their possible cytotoxic effects in men. At present, there is a large request to investigate thee potential acute and chronic effects of nanomaterials especially in vitro in cells. Those studies can provide a mechanistic view on nanomaterial cellular acting. However, the use of proper and relevant bioanalytical methods for evaluating the nanomaterials effects in cells is necessary. 

In this study, we aimed to provide a recent and detailed view on ROS production induced by nanomaterials, especially considering the metalic nanoparticles. In cells, the nanotoxicity can be mediated by a number of substances including ROS. Depending on the composition and shape of a nanomaterial, a variety of ROS can be formed in cells, i.e., O_2_^●−^, ^1^O_2_, ^●^OH, and H_2_O_2_. Thus, the importance of the present review can be recognized in the mechanistic description of a relation of nanomaterials of different chemical compositions and ROS production. We provided the current knowledge of ROS-mediated cellular nanotoxicity together with the possibilities of ROS detection in cells using specific fluorescent probes. In addition, we summarized the detailed description of the relationship between nanomaterials-mediated ROS production and glutathione depletion. Altogether, the prooxidative action of nanomaterials can ultimately lead to the activation of cellular signaling pathways, causing a change in cellular metabolism, cell damage, or even cell death.

## Figures and Tables

**Table 1:** Overview of nanomaterials capable of ROS production [28].

Nanomaterial	Produced ROS	ROS	Half-Life
ZnO [29], SiO_2_ [29], TiO_2_ [30], CuO [31], Ag NPs [32]	Superoxide	O_2_^●^^−^	10^−6^ s
ZnO [33], TiO_2_ [34], CuO [35]	Hydroxyl radical	^●^OH	10^−10^ s
Polystyrene NPs [36], Au NPs [37], TiO_2_ [38], ZnO [39], Ag NPs [40]	Hydrogen peroxide	H_2_O_2_	Stable (x.s, min)
TiO_2_ [41], Ag NPs [42], FeO [43]	Singlet oxygen	^1^O_2_	10^−6^ s

**Table 2:** Overview of fluorescent probes for the detection of ROS [79,95,97,98,114,115,137,141,142,143,145].

Type of ROS	Fluorescent Probe	Excitation/Emission Wavelengths
Superoxide	MitoSox	535/610 nm
	1,3–diphenylisobenzofuran	410/455 nm
Hydroxyl radical	Terephthalic acid	310/420 nm
	Rhodamine nitroxide	560/588 nm
	HKOH-1	500/520 nm
Singlet oxygen	DPAX-1	495/515 nm
	DMAX	495/515 nm
	Singlet Oxygen Sensor Green^®^	504/525 nm
Hydrogen peroxide	2′,7′-dichlorodihydrofluorescein	498/522 nm
	Amplex Red	563/587 nm
	HyPer ratiometric sensor	485/516 nm
	Pentafluorobenzenesulfonyl fluoresceins	485/530 nm
	Europium ion	400/616 nm
	Homovanilic acid	312/420 nm

## Data Availability

Not applicable.
